# Natural extracellular nanovesicles and photodynamic molecules: is there a future for drug delivery?

**DOI:** 10.1080/14756366.2017.1335310

**Published:** 2017-07-14

**Authors:** Katsuyuki Kusuzaki, Takao Matsubara, Hiroaki Murata, Mariantonia Logozzi, Elisabetta Iessi, Rossella Di Raimo, Fabrizio Carta, Claudiu T. Supuran, Stefano Fais

**Affiliations:** ^a^ Department of Musculoskeletal Oncology, Takai Hospital Tenri Nara Japan; ^b^ Department of Orthopaedic Surgery, Mie University Graduate School of Medicine Tsu Mie Japan; ^c^ Department of Orthopaedic Surgery, Matsushita Memorial Hospital Osaka Japan; ^d^ Department of Oncology and Molecular Medicine, National Institute of Health Rome Italy; ^e^ Dipartimento Neurofarba, Sezione di ScienzeFarmaceutiche e Nutraceutiche, Università degli Studi di Firenze Sesto Fiorentino, Florence Italy

**Keywords:** Extracellular nanovesicles, exosomes, photodynamic molecules, acridine orange

## Abstract

Photodynamic molecules represent an alternative approach for cancer therapy for their property (i) to be photo-reactive; (ii) to be not-toxic for target cells in absence of light; (iii) to accumulate specifically into tumour tissues; (iv) to be activable by a light beam only at the tumour site and (v) to exert cytotoxic activity against tumour cells. However, to date their clinical use is limited by the side effects elicited by systemic administration. Extracellular vesicles are endogenous nanosized-carriers that have been recently introduced as a natural delivery system for therapeutic molecules. We have recently shown the ability of human exosomes to deliver photodynamic molecules. Therefore, this review focussed on extracellular vesicles as a novel strategy for the delivery of photodynamic molecules at cancer sites. This completely new approach may enhance the delivery and decrease the toxicity of photodynamic molecules, therefore, represent the future for photodynamic therapy for cancer treatment.

## Exosomes as a drug delivery system

Nanomedicine encompasses old and new technologies to produce nanoparticles with the highest level of efficiency and the lowest toxicity. To achieve this endpoint, the strategic platform on nanomedicine highly recommends the identification of biomimetic nanomaterials. Unfortunately, the clinical investigation on liposome-based drugs, did not show convincing results in term of efficacy and toxicity, probably due to PEGylation^1^. Targeted drug delivery is a promising area that is emerging to improve therapy efficiency, by selectively delivering the drug to target cells, reducing the dose with respect to the equivalent plasma concentrations, and avoiding destruction of non-target tissues. An example is the use of PCSK9-specific siRNA formulated in a lipid nanoparticle to treat metabolic disease in humans, and for which a clinically validated endpoint (i.e. LDL cholesterol) has been obtained^2^. The efficiency of targeted drug delivery is achieved by the attachment of specific ligands to drug delivery vehicles. Nanoparticle size, shape and surface chemistry are also crucial for an efficient delivery to target cells^3^. In contrast, the vast majority of administered liposomes (of uncontrolled size) rather reach the spleen or liver than the target organ or compartment, and the progressive accumulation into the macrophages leads to the high level of toxicity of the liposome-based drugs clinically tested to date. Therefore, it is mandatory to develop methods to produce vesicles with tropism to target organs and controlled size, optimally with diameter <150 nm. Recently, attention was paid to natural nanosized extracellular vesicles (EVs) and/or artificial EV mimics as a state of the art strategy for targeted drug delivery^4^. EVs are nanosized membrane-contained vesicles released in the extracellular space and in biofluids by a variety of cell types^5^ ^,^ ^6^. Natural EVs have been shown to transfer genetic material, proteins, bioactive lipids and other signalling molecules, among cells in a paracrine and systematic manner, thereby mediating intercellular communication in both normal physiological conditions and pathological processes. In the last few years, EVs have emerged as novel putative therapeutic tools for the treatment of various diseases, including cancer^4^ ^,^ ^6–11^.

Whereas cancer-derived EVs apparently promote cancer progression and may cause unwanted effects^12–16^, EVs derived from normal cells have been shown to possess intrinsic therapeutic activity^17–20^. To enhance their therapeutic efficacy, EVs have been loaded with therapeutic agents such as doxorubicin and siRNAs^21–24^. Mesenchymal stem cells (MSC)-derived EVs are proved to be well-tolerated in humans, and in the autologous setting, they are non-immunogenic. Some studies also demonstrate good tolerance in allogeneic and even xenogenic settings^25^ ^,^ ^26^. Therefore, EVs could be superior to viral gene or drug delivery tools, such as VLPs. The demonstration that natural nanovesicles represent the ideal vector for drugs of different natures may thus represent a highly valuable model for nanotechnology. In addition, artificial tuning of EVs or EV mimics have a tremendous potential for their use as drug delivery systems, being immuno-silent or immunoregulatory, and with a specific and directed targeting. From the clinical and translational standpoint, EVs have been seen as potential non-invasive biomarkers for many diseases. Many of these studies contain very useful information about the composition and disease-related changes that may reveal important targets for therapeutic intervention.

Cancer has sadly to be considered an unmet clinical need being unbearable the amount of deaths yearly worldwide (9–10 million). In fact, despite the recent developments of targeted therapies against cancer over-expressing targets, overall survival remains low, and the development of less toxic and efficient drug delivery tools represent an urgent unmet medical need. Likely, if this is accomplished, cancer-specific drugs could reach the tumour in higher doses and improve clinical effectiveness.

Natural nanovesicles (exosomes) were proven to be able to deliver anti-cancer drugs^27^ and in this issue the ability of human exosomes to deliver photodynamic molecules has been clearly shown^28^. This is an important achievement inasmuch as the use of photodynamic molecules may well represent the future of cancer therapy for their property to concentrate into tumour tissues and only there activable through either fluorescent light or X-rays^29^.

## Photodynamic molecules and cancer

### Principle of photodynamic therapy

**Figure 1.**
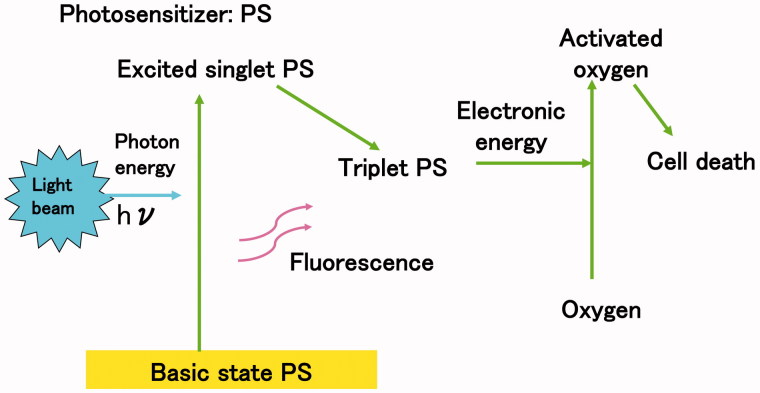
Energy transfer in photodynamic therapy. Following a light beam, the photosensitizer reaches an excited singlet state and moves to a triplet excited state. The excited triplet photosensitizer reacts directly with oxygen through energy transfer generating activated oxygen.

Photodynamic therapy (PDT) has been used to efficiently kill cancer cells and represent a well-established and alternative treatment modality for the treatment of different types of cancers. In PDT, a photosensitizer (usually a photo-reactive agent) and a source of light (photon) beam are needed. Photosensitizer accumulates into cancer cells and light beam is irradiated to kill them. When the photosensitizer is excited by a light beam, an energy of photon (hν) transfers to photosensitizer, moving it from a basic singlet state to an excited triplet state (Figure 1). Since excited photosensitizer having high energy electron is very unstable, it also rapidly transfers electrons to oxygen localised in cytoplasm to produce activated oxygen (Figure 1).

**Figure 2.**
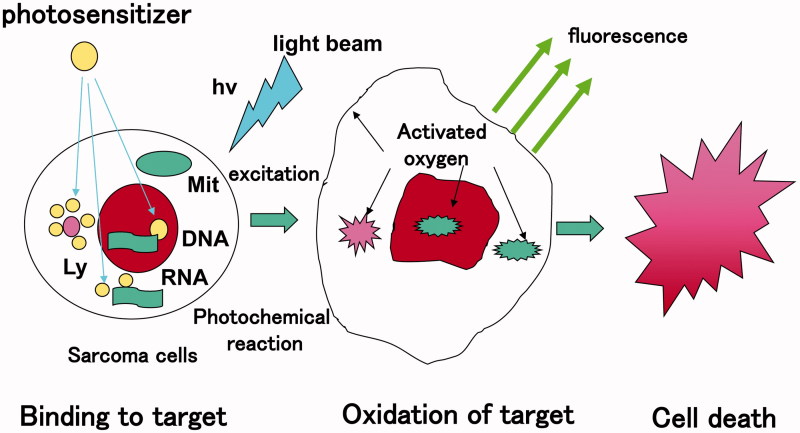
Mechanism of cytocidal effect of photodynamic therapy. Irradiation with light beam induces the formation of activated oxygen through energy transfer. Activated oxygen is highly reactive and cytotoxic. It reacts with biomolecules (i.e. lipids, proteins, and nucleic acids of cellular or lysosomal origin) inducing cell death through activation of the apoptotic pathway.

Activated oxygen which behaves as a free radical, oxidises proteins and fatty acids of cellular or lysosomal membrane to cause apoptosis through membrane rupture (Figure 2).

Most photosensitizers emit fluorescence at the moment when they return from excited to basic state. The wavelength of this fluorescence is always longer than the excited light. There are various kinds of photosensitizers. Most of them are coloured dyes, such as derivatives of acridines^29^ ^,^ ^30–37^, flavins phenothiazines^38–49^, quinolones^50–60^, cyanines^49^ ^,^ ^61–69^, and biological compounds of hematoporphyrin (Hp) and its precursors, like porphyrin (Pf)^70^, 5-amino levulinic acid (ALA)^71–73^, etc. The ideal photosensitizer should: (i) accumulate specifically in cancer cells, sparing normal cells; (ii) kill only cancer cells after light beam irradiation and (iii) be not toxic for the human body, even after irradiation. To improve specific accumulation of photosensitizer, some new technologies of delivery system for PDT were lately reported. One approach is using nanoparticle loaded with indocyanine green^74^, another is using cancer-specific antibody conjugated with IR700, a specific photosensitizer^75^. These two approaches are now ongoing on under clinical application. Exosomes described here represent a novel option of delivery systems of photodynamic molecules, with the final goal to efficiently increase the cancer-specific accumulation rate.

Although there are many light sources for PDT, both xenon lamp and laser system are available for clinical application. Laser has stronger power to excite photosensitizer, but is much more expensive compared with xenon lamp.

PDT originated from discovery of the phototoxic effect of Acridine Orange (AO) on protozoa by a doctoral student, Oscar Raab, in 1900^76^. At present, PDT using a Hp or its precursor, like Pf and ALA, with a laser beam is one of established modalities for cancer therapy, especially for early-stage superficial cancers of the skin, lung, oral cavity, oropharyngeal tract, oesophagus, gastrointestinal tract, urinary bladder, etc.^77^.

### PDT with AO (AO-PDT)

AO was first extracted from coal tar in Germany in the late 19th century, as a weak basic dye for staining of clothes or microorganisms^78^, and has many unique biological activities, such as antitumor activity^79–85^, photosensitising activity^76^ ^,^ ^86–88^, pH detecting activity^89^, and fluorescence detection or toxic activity in sperm^90^, bacteria^91^ ^,^ ^92^, viruses^93^, parasites, especially the malaria parasites^94^ ^,^ ^95^, and fungi^96^. AO emits green (533 nm) or orange (656 nm) fluorescence following blue light (492 nm) excitation. Since AO has a very low molecular weight (MW 265), it has the capability to rapidly flow into the cytoplasm through the plasma membrane binding to the DNA, RNA^97^ ^,^ ^98^) and acidic lysosomes^99^ ^,^ ^100^. AO selectively accumulates in cancer cells, especially in acidic lysosomes, emits fluorescence after blue excitation, and kills cancer cells via apoptosis by activated oxygen.


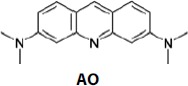


**AO**

Although it is well known that AO is mutagen for bacteria^91^ ^,^ ^92^, there is no evidence to prove that AO is carcinogen for mammalians including human^101^ ^,^ ^102^. Our study using mice revealed that LD_50_ of AO intravenously administrated was 28–30 mg/kg (clinical use: 1 mg/kg, local administration)^103^.

### Clinical application of AO-PDT

AO-PDT has been exploited by the Kusuzaki’s group in cancer therapy. Local administration of AO and irradiation of blue light from a xenon lamp after minimal invasive surgery has been used in treatment of patients with musculoskeletal sarcomas to avoid wide resection which causes serious limb dysfunction. More than 200 patients have been treated with AO-PDT over 10 years and its clinical outcome showed (i) low risk of local recurrence which is almost the same that with conventional wide resection and (ii) superior limb function compared with that by wide resection^29^ ^,^ ^31–37^.

A recent study published in this journal^28^ has clearly shown that AO delivered by natural nanovesicles (exosomes) released by human normal cells such as monocytes/macrophages highly increase its uptake by tumour target cells and its efficacy as cytotoxic molecule. This result is of course highly promising for the use of AO in cancer treatment with either local or systemic approaches. Furthermore, a sulphonamide derivative of AO which has been recently reported^104^, was shown to act as a low nanomolar carbonic anhydrase CA^105^ ^,^ ^106^ inhibitor against the tumour-associated isoforms CA IX and XII; making it an interesting candidate both for PDT as well as EV formulations. Work is in progress in our laboratories for evaluating this interesting drug candidate for possible applications in targeting hypoxic tumours.

## Conclusions

PDT is a promising alternative approach for the treatment of cancer due to its selective ability to kill tumour cells sparing normal cells. It involves a photosensitizer that is activated by light of a specific wavelength, which induces cell death in target cells in turn leading to the destruction of tumour cells. Unfortunately, the clinical application of PDT is limited by the side effects elicits by systemic administration of the photosensitizers.

Drug delivery is probably as important as drug design, although only in the last period this started to be seriously taken into consideration by the drug industries and academic community. Exosomes and EVs may have a crucial role in such processes due to reasons highlighted in this paper. Loading EVs with various drugs, including AO and similar agents used in PDT (Table 1)^30–37^ ^,^ ^44^ ^,^ ^63^ ^,^ ^66–69^ ^,^ ^108?143^, may lead to an enhanced delivery, decreased toxicity and diminished side effects. The recent example of EVs loaded with AO from Fais’s group^28^ clearly indicates that this is the future in PD therapy.

**Table 1.** A list of photodynamic molecules used in PDT.

Compound	Structure	References
Acai oil		^107^
Acridine orange (AO)		^29^
5-Aminolevulinic acid (ALA)		^109–111^
Chlorins		^112–115^
C-Phycocyanin		^116^
Cyclodextrin		^117^^,^^118^
Coumarin derivative		^119^^,^^120^
Curcumin		^121^^,^^122^
DPAO2		^123^
DPP-ZnP/DPP-ZnP-DPP		^124^
Erythrosine		^125^
Folate–albumin–photosensitizer conjugate		
Hypericin		^126–130^
Methylene blue (MB)		^43^^,^^131^^,^^132^
PdTPPo/TPPo		^133^
Phthalocyanines		^62^^,^^65^^,^^66^^,^^68^
PPaN-20		^134^
Porphyrin		^135–141^
PyP/yPyyPy/porphycenes		^142^
Quinone		^143^

Moreover, Acridine Orange is a clear example of a molecule that works as both tracer (being fluorescent) and as anti-tumour drug, thus representing a clear example of a molecule with a theranostics potential. Exosomes may be extremely helpful in future strategies aimed at delivering much better to the disease’s sites either old or new therapeutic molecules or even molecules with both diagnostic and therapeutic actions, with therefore theranostics properties. Between these molecules will be of course included all the known compounds with photodynamic properties as well.
